# Investigation of Phytochemical Composition, Antioxidant and Antibacterial Activity of Five Red Flower Extracts

**DOI:** 10.3390/antiox14020151

**Published:** 2025-01-27

**Authors:** Diana Ionela Popescu (Stegarus), Oana Romina Botoran, Ramona Maria (Iancu) Cristea

**Affiliations:** 1 National Research and Development Institute for Cryogenic and Isotopic Technologies—ICSI Ramnicu Valcea, 240050 Ramnicu Valcea, Romaniaoana.dinca@icsi.ro (O.R.B.); 2 Department of Agricultural Science and Food Engineering, “Lucian Blaga” University of Sibiu, 7-9 Dr. Ion Ratiu, 550024 Sibiu, Romania

**Keywords:** red flowers, polyphenols, anthocyanins, phenolic compounds, antioxidant activity, antibacterial activity

## Abstract

The presence of bioactive compounds in plants with red flowers constitutes a valuable natural resource for the food, pharmaceutical, and medical industries. This paper aims to highlight these characteristics to facilitate the selection of extracts according to market needs. The analyzed parameters can provide valuable insights into the compositional variability of the five red flower plants, as new sources of bioactive compounds and natural antioxidants of plant origin. Five plants with red flowers (*Magnolia liliiflora*, *Chrysanthemum morifolium*, *Hibiscus sabdariffa*, *Petunia grandiflora*, *Rosa hybrida* L.) were selected and investigated regarding their polyphenol concentration, total anthocyanins, antioxidant activity, phenolic compounds, and antimicrobial activity. Total polyphenols were found in concentrations ranging from 75.17 ± 0.16 mgGAE/g (*C. morifolium*) to 107.49 ± 0.19 mgGAE/g (*R. hybrida* L.), and total anthocyanins between 14.99 ± 0.13 mg Cy-3-Glu/g and 21.66 ± 0.18 mg Cy-3-Glu/g. The antioxidant activity DPPH/IC50 mg/mL for the extract from *R. hybrida* L. flowers reached a value of 29.35 ± 0.17 mg/g, with ABTS and FRAP achieving maximum values of 49.89 ± 0.24 mmol TE/g and 48.68 ± 0.23 mmol TE/g, respectively. The antibacterial activity was particularly evident against strains commonly encountered in human pathology, *Escherichia coli* and *Staphylococcus aureus*, with reduced or no impact on strains of *Pseudomonas fluorescens* and *Proteus mirabilis*. The results demonstrated that the extracts from the selected, red-flowered plants contain valuable biologically active compounds in generous quantities, thus recommending their use in the food, pharmaceutical, and medical sectors.

## 1. Introduction

Plant extracts are currently a topic of increasing study; they are incorporated into natural medicines that are more easily assimilated by the human body. Medicinal plants have long been considered the most critical sources for obtaining natural antioxidants, but more studies are leading to the investigation of other natural sources such as petals (calyces), leaves, or flower stems. It is also noted that commercial antibiotics are losing effectiveness in the presence of increasingly resistant microorganisms, suggesting that the use of appropriate natural extracts could resolve many medical situations. Numerous studies focus on the efficacy of plant extracts against bacteria, particularly those with frequent human impact [1], like *Escherichia coli* (*E. coli*). The authors of [2] evaluated the antimicrobial effects of the ethanolic extract from the leaves of five plants—guava (*Psidium guajava*), sage (*Salvia officinalis*), rhamnus (*Ziziphus spina Christi*), mulberry (*Morus alba* L.), and olive (*Olea europaea* L.)—on both Gram-positive and Gram-negative bacteria, with significant results (MIC; MBC between 625 μg/mL to 5000 μg/mL). To appreciate their curative properties, a rigorous analysis of various bioactive compounds with pharmaceutical, medical, and food potential is essential [3,4,5,6,7]. In plants, various types of flavonoids such as anthocyanins, flavones (luteolin, apigenin), isoflavones, flavonols (rutin, quercetin), and flavanones have been identified. It is known that the polyphenols present in plants lead to antioxidant properties with therapeutic effects, the quantity of the extract being directly proportional to the method and solvent used [8]. An important component of the polyphenol composition are anthocyanins. These are responsible for the color of flowers or fruits, with pigments ranging from pink to red or purple, blue or violet. Existing methods can showcase the pigments and the color palette of petals or fruits [9,10,11,12].

Anthocyanins are glycosides of anthocyanidins, with aglycones at their base forming the flavylium cation. This consists of a benzopyrylium nucleus and a phenolic ring. All anthocyanidins contain hydroxyl groups: typically at positions 3, 5, 7 on the benzopyrylium nucleus, and at positions 3′, 4′, 5′ on the phenolic ring. The most commonly encountered anthocyanin elements in plants are pelargonidin, cyanidin, delphinidin, peonidin, petunidin, and malvidin in their glucosyl-rhamnosyl-glucoside or glucosyl-rhamnoside forms [13]. Plant anthocyanins can exhibit antimicrobial, anticarcinogenic, and antidiabetic properties, contributing to the treatment of various medical conditions [14,15,16,17,18]. Studies have demonstrated their presence in edible flowers, with the extracts obtained serving as a varied basis also for the food industry [18]. Depending on pedoclimatic and agronomic factors, species, or technological conditions, the content of polyphenols/anthocyanins can vary substantially in plants [19,20]. Thus, magnolia flowers have demonstrated significant medicinal capabilities, being used in the treatment of spastic conditions as vasodilators, in states of diarrhea, anxiety, being hepatoprotective, and anticarcinogenic [21,22,23]. *Magnolia liliiflora* (*M. liliiflora*) is one of the most widely found species in all regions with a suitable climate, originating from China and Japan. It is acclimatized both in North America and Europe, being considered an ornamental plant, with flower colors varying from pink to violet. *Chrysanthemum morifolium* (*C. morifolium*) is widespread in numerous areas of the globe, the flowers being rich in antioxidant compounds, especially flavonoids [24].

Extracts and teas possess calming and sedative properties [25] and antibacterial qualities, being used in treating arthritic and inflammatory conditions [20,26,27,28,29,30]. Extracts from *H. sabdariffa* (*H. sabdariffa*) flowers exhibit potential antioxidant and antimicrobial properties, contributing to the regulation of cholesterol and triglycerides in the body, including anticarcinogenic and antidepressant qualities [31,32]. Valuable bioactive compounds have also been identified in the extracts of *Petunia grandiflora* (*P. grandiflora*) and *Rosa hybrida* L. (*R. hybrida* L.) flowers, with potential antioxidant, anticarcinogenic, or antimicrobial effects [33,34,35,36]. This study aims to evaluate the bioactive compounds from five plants with red flowers, the antioxidant and antibacterial capacities of the extracts from *M. liliiflora*, *C. morifolium*, *Hibiscus sabdariffa* (*H. sabdariffa*), *P. grandiflora*, and *R. hybrida* L., with the goal of providing a body of useful information to the pharmaceutical, medical, and food industries

## 2. Materials and Methods

### 2.1. Materials

Extracts from the flowers of *M. liliiflora*, *C. morifolium*, *H. sabdariffa*, *P. grandiflora*, *R. hybrida* L., reagents, microbial strains, and culture media were used. The flowers were harvested between April and October 2023, identified and documented by specialized personnel at the CCBIA Research Center of the Faculty of SAIAPM/ULB Sibiu, under voucher no. 398/1-398/5. The following analytical purity reagents from Sigma-Aldrich GmbH, Steinheim, Germany were utilized: Folin–Ciocalteu reagent, sodium carbonate, acetic acid, 1N hydrochloric acid, acetonitrile, DPPH (2,2-diphenyl-1-picrylhydrazyl), Trolox (6-hydroxy-2,5,7,8-tetramethyl-chroman-2-carboxylic acid), ABTS (2,2′-azino-bis-3-ethylbenzothiazoline-6-sulfonic acid diammonium salt), TPTZ (2,4,6-tripyridyl-s-triazine), ethanol (99.8%), methanol (>99.9%), gallic acid (>99%), cyanidin-3-glucoside (>99%), chlorogenic acid (96%), ferulic acid (95%), p-coumaric acid (98%), caffeic acid (98%), m-coumaric acid (98%), sinapic acid (98%), trans-cinnamic acid (98%), benzoic acid (98%), ellagic acid (99%), p-hydroxybenzoic acid (99%), rosmarinic acid (98%), syringic acid (98%), vanillic acid (98%), rutin (95%), (+)-catechin (98%), quercetin (95%), luteolin (95%), and apigenin (95%).

#### Antibacterial Activity

Antibacterial testing was conducted on the following reference strains: *S. aureus* ATCC 33862, *E. coli* ATCC 25922, *P. fluorescens* ATCC 13525, *P. mirabilis* ATCC 12453, using standard 10 μg Ampicillin discs (Thermo Scientific™ Oxoid™, Waltham, MA, USA) and Ampicillin sodium solution 50 mg/mL, with a working concentration of 20 μg/mL as control. Mueller–Hinton agar and Mueller–Hinton broth from Sigma-Aldrich GmbH, Steinheim, Germany, were utilized as culture media.

### 2.2. Methods

Flowers from *M. liliiflora*, *C. morifolium*, *H. sabdariffa*, *P. grandiflora*, and *R. hybrida* L. were dried for three days at a temperature of 42 ± 0.5 °C until a constant mass was achieved. The dried material was ground to a particle size ranging between 200–500 μm. A quantity of 250 g of dried material was homogenized with 250 mL of a methanol/water solution (70:30 *v*/*v*). The decanted samples were filtered (Whatman no. 4) and concentrated using a rotary evaporator. The extractions were repeated three times. To report the efficiency of the extractions, the following formula was used, where R = weight of extracted plant residues, and S = weight of plant raw sample:

Extract yield (%) = R/S × 100(1)

#### 2.2.1. Determination of Total Polyphenols and Antioxidant Activity

Total polyphenols were identified using a slightly modified Folin–Ciocalteu method [37]. The extract, in a volume of 0.20 mL, was homogenized with 0.80 mL of Folin–Ciocalteu reagent (10% *v*/*v*) and 1 mL of sodium carbonate (7.5% *m*/*v*). After incubation in the dark for one hour at room temperature, readings were taken at a wavelength of 750 nm using the UV-1900 SHIMADZU spectrophotometer (Shimadzu Corporation, Kyoto, Japan). Results were expressed in milligrams of gallic acid equivalent per gram of dry substance (mg GAE/g). Antioxidant activity was determined using three methods: DPPH, ABTS, and FRAP, with readings performed on the UV-1900 SHIMADZU spectrophotometer (Shimadzu Corporation, Kyoto, Japan) [38,39].

DPPH: The method involves preparing a stock methanolic solution of DPPH with a concentration of 600 μM, which is stored at –20 °C. From this, working methanolic solutions with a concentration of 60 μM are prepared. A mixture of 25 μL of the sample with 175 μL of the DPPH working solution is allowed to react for 30 min at a temperature of 20 °C. Following this reaction period, the absorbance is measured at a wavelength of 517 nm. A control sample is prepared similarly, without the extract, using only distilled water. A calibration curve with Trolox is established, and the results are expressed as μM of Trolox equivalent per mg of dry substance.

The percentage of inhibition (*I*) is calculated according to the equation:

*I* (%) = [(*Abs*_*A*0_ − *Abs_sample_*):*Abs*_*A*0_] × 100 (2)

where *Abs_A_*_0_ is the absorbance value of the control sample, and *Abs_sample_* is the absorbance value of the test sample.

FRAP: A quantity of 50 mL of acetate buffer (300 mM/pH = 3.6), 5 mL of FeCl_3_ (20 mM), and 5 mL of TPTZ solution (10 mM) acidified with 150 μL HCl were combined to prepare the FRAP solution. In a container, 0.1 mL of extract, 0.5 mL of FRAP solution, and 2 mL of pure water were blended, and incubated in the dark for one hour. A Shimadzu UV 1900 spectrophotometer was used to measure the extinction at λ = 595 nm. A linear calibration curve (0.15–0.5 μmol Trolox/mL) was derived, and the results expressed as μmol TE/g d.w.; ascorbic acid was used as a control.

ABTS: A stock solution of potassium persulfate (2.45 mM) and diammonium salt of 2,2′-azinobis [3-ethylbenzothiazoline-6-sulfonic acid] (7 mM) was prepared, then kept in the dark at room temperature for 16 h. The stock solution was diluted so that the extinction (λ = 734 nm) was 0.70 ± 0.02. In a container, 20 μL of extract was added to 2 mL of the diluted stock solution and blended for 30 s. After exactly one minute, the extinction at λ = 734 nm was measured using a spectrophotometer. A linear calibration curve (0.125–2.0 μmol Trolox/mL) was derived, and the results expressed as μmol TE/g d.w.; ascorbic acid was used as the positive control.

#### 2.2.2. Determination of Total Anthocyanins

Total anthocyanins were determined from extracts of *M. liliiflora*, *C. morifolium*, *H. sabdariffa*, *P. grandiflora*, and *R. hybrida* L. using the spectrophotometric method described by Lee et al. (2005), which is based on pH difference [40]. The method involves homogenizing 250 μL of each extract with 750 μL potassium chloride (KCl) at pH 1.0 and sodium acetate (CH_3_CONa) at pH 4.5. The samples were incubated for 30 min at a temperature of 23 °C. Absorbance was measured at 515 and 700 nm using the UV-1900 SHIMADZU spectrophotometer (Shimadzu Corporation, Kyoto, Japan). *Absorbance* (A) was calculated as follows:

*A =* (*Abs*_515nm_
*− Abs*_700nm_)*_pH_*_1.0_ − (*Abs*_515nm_
*− Abs*_700nm_)*_pH_*_4.5_ (3)

Total *Anthocyanins* were determined according to the equation:

*Anthocyanins* (mg Cy-3-glu/L) = *A* × *MW* × *DF* × 10^3^/*ε* × *l* (4)

where *A* = absorbance, *MW* (molecular weight) = 449.2 g/mol for Cy-3-glu, *DF* = dilution factor, *l* = path length = 1 cm, *ε* = 26,900 molar extinction coefficient for cyanidin-3-glucoside, L × mol^−1^ × cm^−1^, 10^3^ = conversion factor from g to mg.

Results were converted to milligrams of cyanidin-3-glucoside equivalent per gram of dry extract (mg Cy-3-glu/g d.w.).

#### 2.2.3. Identification of Phenolic Compounds by HPLC-DAD Method

Phenolic compounds in the extracts from *M. liliiflora*, *C. morifolium*, *H. sabdariffa*, *P. grandiflora*, and *R. hybrida* L. were determined using a fully equipped Agilent 1200 HPLC-DAD system (Agilent Technologies, Santa Clara, CA, USA) and a C18 chromatographic column (Zorbax SB-Aq: 250 mm × 4.6 mm i.d., 5.0 μm particle size). The injected sample volume was 10 μL, with eluent A being a solution of water-acetic acid (95/5 *v*/*v*) and eluent B acetonitrile/water/acetic acid (100/95/5 *v*/*v*/*v*), following a gradient elution program according to the method described by Cristea [38].

#### 2.2.4. Determination of Antimicrobial Activity: Diffusion Method

To establish the antimicrobial activity, a modified Kirby–Bauer method was used, which involves activating the bacteria for 24 h in Mueller–Hinton broth and preparing bacterial suspensions at a concentration of 0.5 McFarland (density 0.5 McF = 1.5 × 10^8^ CFU/mL).

In Petri dishes containing Mueller–Hinton culture medium, the bacterial culture was deposited and 6 mm diameter discs were placed.

These discs were impregnated with 10 μL of the extracts being tested, then incubated for 24 h at 37 °C.

The inhibition zone, including the diameter of the disc (mm), was measured. Each test was performed in triplicate, resulting in an average that was reported. Standard ampicillin discs (10 μg) were used as control samples.

##### Determination of Minimum Inhibitory Concentration (MIC) and Minimum Bactericidal Concentrations (MBC)

The method used is the one described by Ibrahim [41], slightly modified, which involves creating a series of seven successive dilutions of extracts at concentrations of 40 mg/mL, 20 mg/mL, 10 mg/mL, 5 mg/mL, 2.5 mg/mL, 1.25 mg/mL, and 0.625 mg/mL. Bacterial strains *S. aureus*, *E. coli*, *P. fluorescens*, and *P. mirabilis* were activated for 24 h in MHB culture medium. A volume of 1 mL of the diluted extract, prepared according to the established dilution order, was introduced into test tubes. Subsequently, 0.1 mL of the bacterial strain (density 0.5 McF = 1.5 × 10⁸ CFU/mL) was added, mixed thoroughly, and incubated at 37 ± 0.5 °C in an incubator. After 18 h, the sets of tubes were observed. Turbidity was visually observed and then verified spectrophotometrically. Based on the dilution and the degree of turbidity, the MIC was determined. Tubes that showed no visible microbial growth (turbidity) were tested to determine the MBC by inoculation on solid MH medium. All plates were then incubated at 37 °C for 24 h. After incubation, the MBC was recorded as the concentration at which no visible growth was observed. The determinations were performed in triplicate.

#### 2.2.5. Statistical Analysis

The statistical analysis focused on the covariance and correlations existing between the studied extracts and the identified phenolic compounds. Pearson correlations (*p* < 0.05 and *p* < 0.01) were used to identify relationships among all variables included in the dataset. The software Addinsoft XLSTAT version 2014.5.03 (Addinsoft Inc., New York, NY, USA) was utilized to perform the statistical analyses.

## 3. Results

Reviewing the results obtained in Table 1, it is observed that *M. liliiflora* has an average dry extract weight of 55.09 mg, with individual values ranging between 49.87 mg and 61.27 mg, and an average extract concentration of 5.50 mg/mL, varying between 4.98 mg/mL and 6.12 mg/mL.

*C. morifolium* is noted for an average extract weight of 40.45 mg, with values between 37.99 mg and 43.23 mg and a concentration of 4.04 mg/mL, ranging from 3.79 mg/mL to 4.32 mg/mL.

From *H. sabdariffa* flowers, the average dry extract measured was 45.18 mg, with values ranging between 44.16 mg and 45.81 mg, and an extract concentration of 4.51 mg/mL. *P. grandiflora* and *R. hybrida* L. are notable for an average dry extract of 29.20 mg and 71.09 mg, respectively, with concentrations of 2.92 mg/mL and 7.10 mg/mL. Three extractions were performed from each plant, which were individually analyzed and subsequently homogenized; the final resulting extracts were then processed. Table 2 contains information about the antioxidant activity and the content of bioactive compounds (particularly antioxidants) in five plant species: *M. liliiflora*, *C. morifolium*, *H. sabdariffa*, *P. grandiflora*, and *R. hybrida* L. Values are presented for the following parameters:

TPC (Total Phenolic Content): The total content of phenolic compounds, measured in mg GAE (gallic acid equivalents) per gram of dry weight (d.w.).

Total Anthocyanin Content: Measured in mg Cy-3-Glu (cyanidin-3-glucoside) per gram of dry weight.

Antioxidant Activity: Measured by the DPPH assay (free radical scavenging capacity), expressed as IC50 mg/mL (the concentration necessary to inhibit 50% of free radicals).

Antioxidant Activity by ABTS Test: Expressed in μmol TE (Trolox equivalents) per gram of dry weight.

Antioxidant Activity by FRAP Test (Ferric Reducing Antioxidant Power): Expressed in μmol TE per gram of dry weight.

Depending on the plant species, the following results were observed: *R. hybrida* L. shows the highest values for phenolic compound content (TPC: 107.49 mg GAE/g d.w.) and anthocyanins (total anthocyanins: 21.66 mg Cy-3-Glu/g d.w.). It also has the highest antioxidant activity in the ABTS (49.89 μmol TE/g d.w.) and FRAP (48.68 μmol TE/g d.w.) tests. This suggests that *R. hybrida* L. has the greatest antioxidant capacity among the species analyzed. *M. liliiflora* also has high values, with a phenolic content of 94.34 mg GAE/g d.w. and an anthocyanin content of 18.24 mg Cy-3-Glu/g d.w. Its antioxidant activity is good, but lower than that of *R. hybrida* L., in the ABTS (42.19 μmol TE/g d.w.) and FRAP (45.71 μmol TE/g d.w.) tests. *C. morifolium* and *H. sabdariffa* have relatively similar values, both exhibiting moderate phenolic and anthocyanin contents.

Reviewing the results from Table 3, it is noted that *M. liliiflora* contains a total concentration of compounds of 23,291.35 μg/mL with notable amounts of chlorogenic acid (5683.35 μg/mL), rutin (3456.32 μg/mL), syringic acid (893.27 μg/mL), and quercetin (892.88 μg/mL). Certain acids, such as ellagic, rosmarinic, salicylic, and sinapic acids, are absent. *C. morifolium* registers a significant total concentration of 21,710.25 μg/mL and is distinguished by a high amount of benzoic acid (11,209.12 μg/mL) and gallic acid (562.55 μg/mL), as well as rutin (2996.48 μg/mL). It contains a diversity of compounds, but lacks sinapic acid and trans-cinnamic acid. *H. sabdariffa* is the species with the highest total compound concentration at 25,309.49 μg/mL. It stands out for benzoic acid (12,009.37 μg/mL) and ellagic acid (234.77 μg/mL), and notable presence of rutin and quercetin (1999.57 μg/mL and 1001.72 μg/mL, respectively), as well as luteolin and apigenin. *P. grandiflora* has a total compound concentration of 18,435.82 μg/mL. It is rich in benzoic acid (10,568.93 μg/mL) and rutin (2341.73 μg/mL). It lacks ellagic, rosmarinic, and sinapic acids, but contains a variety of other phenolic compounds. *R. hybrida* L. records the highest total compound concentration alongside *H. sabdariffa*, at 25,578.06 μg/mL. It has the highest concentrations of benzoic acid (13,421.74 μg/mL) and syringic acid (1231.55 μg/mL), as well as a significant presence of gallic acid, rutin, and quercetin. It contains nearly all the compounds mentioned in the table, except for sinapic acid.

**Figure 1:**
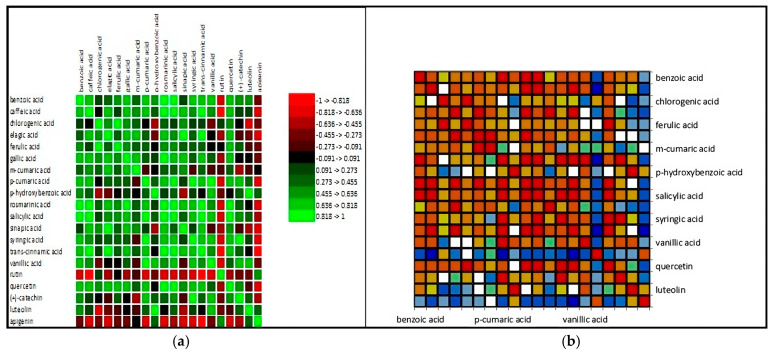
Correlation matrix of (**a**) the phenolic compounds and (**b**) the correlation map resulting from the analyses performed on the five extracts from red flowers (*M. liliiflora*, *C. morifolium*, *H. sabdariffa*, *P. grandiflora*, *R. hybrida* L.). The distribution of compounds shows that most plants exhibit significant concentrations of acids and phenolic compounds with variations depending on the species. Comparing between species, it can be stated that the *R. hybrida* species exhibits the highest concentrations in several compound categories, indicating a high biochemical potential compared to the other species (Figure 2).

Analyzing the data from a statistical perspective, it is notable that the extracts from red flowers are rich in bioactive elements, with their value being specific to each plant. Figure 1 displays the correlation matrix between the analyzed compounds and the map of this correlation.

**Figure 2:**
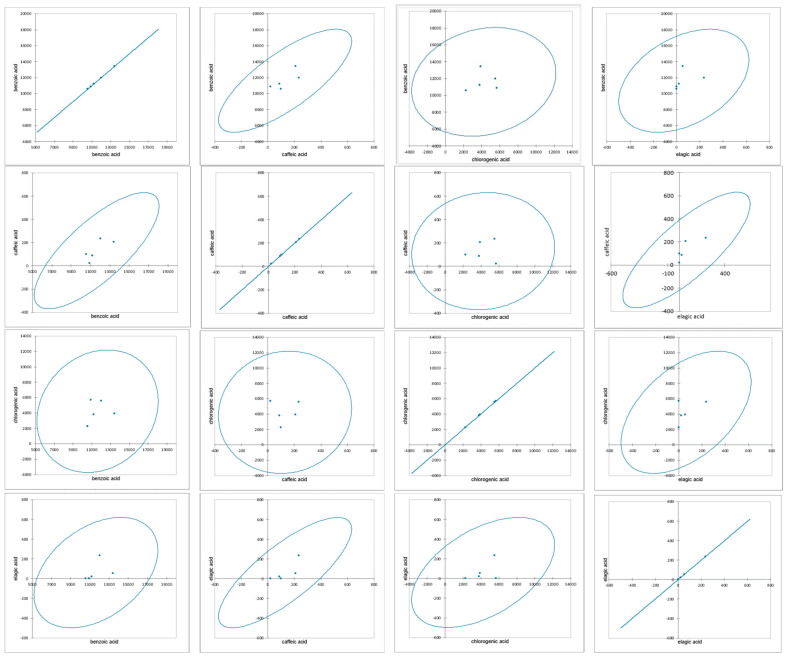
Covariation and correlations between the main phenolic compounds identified in the five extracts from red flowers (*M. liliiflora*, *C. morifolium*, *H. sabdariffa*, *P. grandiflora*, *R. hybrida* L.).

Statistical analysis demonstrated significant correlations between the main phenolic compounds evaluated in the five extracts under study. The potential and importance of the extracts, as well as their configuration within the system, were statistically validated and supported as a result of the analyses performed. The samples exhibited significant species variations, providing an overall visual representation of inter-varietal patterns of similarity or difference, depending on the investigated characteristics.

Table 4 presents the antibacterial activity of various plant extracts against several bacterial strains, compared to ampicillin. Four bacterial species were tested: *S. aureus* ATCC 33862, *E. coli* ATCC 25922, *P. fluorescens* ATCC 13525, and *P. mirabilis* ATCC 12453 using extracts from *M. liliiflora*, *C. morifolium*, *H. sabdariffa*, *P. grandiflora*, *R. hybrida*, and Ampicillin (positive control). Against *S. aureus* ATCC 33862, all extracts displayed antibacterial activity, notably *M. liliiflora* and *H. sabdariffa* (++++) similar to *R. hybrida* (+++) and Ampicillin (+++++). In terms of MIC and MBC, the most effective extracts are from *M. liliiflora* and *H. sabdariffa* (5 mg/mL and 10 mg/mL, respectively) compared to ampicillin (<1.5 μg/mL). All extracts are active (++ or +++) with ampicillin being the most effective (++++) against *E. coli* ATCC 25922, having a MIC of 10 mg/mL and an MBC of 20 mg/mL for *M. liliiflora*, *H. sabdariffa*, and *R. hybrida*. For the strain *P. fluorescens* ATCC 13525, the antibacterial activity of extracts from *M. liliiflora*, *C. morifolium*, and *H. sabdariffa* shows weak activity (+), while *P. grandiflora* and *R. hybrida* show no activity (−). All active extracts have a weak MIC (40 μg/mL), far from ampicillin (<1.5 μg/mL), and very high or ineffective MBC values (>40 μg/mL), establishing their inactivity against *P. mirabilis* ATCC 12453. The extracts from *M. liliiflora* and *H. sabdariffa* are noted for high efficacy, being the most effective extracts against most bacteria. *P. fluorescens* is the most resistant to the tested extracts. Although the plant extracts have shown antibacterial activity, their efficacy is significantly lower compared to ampicillin. *M. liliiflora* and *H. sabdariffa* hold the greatest potential for use in future research for the development of antimicrobial agents.

## 4. Discussion

*M. liliiflora* is noted for a moderate amount of dry extract and an average concentration of it. In comparison, flowers from *C. morifolium* yield a smaller amount of extract resulting in a lower concentration. The quantity and concentration of the extract from *H. sabdariffa* are relatively uniform and moderate. *P. grandiflora*, however, shows the lowest values both in terms of extract quantity and concentration compared to the other species. Studies conducted to date show that both the quantity and concentration of extract from flowers largely depend on the applied methods as well as the solvents used [42,43,44]. Observing the Total Phenolic Content expressed in mg GAE/g d.w. (mg gallic acid equivalent per gram of dry weight), it is noted that *R. hybrida* L. has the highest phenolic content followed by *M. liliiflora*. The species with the lowest phenolic content is *P. grandiflora*, this classification being valid also for total anthocyanins. Other studies show that extracts from red flowers present significant values of polyphenols [45]. TPC values range from 17.35 to 21.73 mg RE/g for Hibiscus. Park et al. (2018) obtained TPC values for magnolia flowers of 16.94 mg/g, and TAC of 0.78 mg/g [46]. Anthocyanins are the most relevant compounds that contribute to the antioxidant capacity of plants. Significant results were obtained by Ogata et al. (2005), Saati et al. (2018) (rose), Griesbach et al. (1999) (petunias), Li et al. (2022) (lilies), Hoang et al. (2023), Lestyan et al. (2014), Ademiluyi et al. (2013) (121.5 mg Cyanidin-3-rutinoside equivalent/100 g, H. sabdariffa), Amer et al. (2022), Grajeda-Iglesias et al. (2016) (hibiscus), Shi et al. (2015) (magnolia), Vankar et al. (2010) (*Canna indica*, *Clitoria ternatea*, *Delonix regia*, *Hibiscus mutabilis*, *Impatiens balsamina*, *Ixora chinensis*, *Jatropha integerrima*, *Lagerstroemia indica*, *Mirabilis jalapa*, *Nerium oleander*, *Portulaca graniflora*, *Quisqalis indica*, *Rosa indica*, *Ruellia tuberosa*, and *Thunbergia erecta*, with TAC values ranging from 36.22 mg/kg to 338.61 mg/kg) [47,48,49,50,51,52,53,54,55,56,57].

*R. hybrida* L. exhibited the best antioxidant capacity (IC50 = 29.35 mg/mL), followed by *M. liliiflora* (23.11 mg/mL) and *H. sabdariffa* (22.05 ± 0.12 mg/mL). The maximum antioxidant activities for ABTS (μmol TE/g d.w.) and FRAP (μmol TE/g d.w.) are observed in extracts from *R. hybrida* L. However, *H. sabdariffa* shows slightly higher antioxidant activity than *C. morifolium* in both ABTS and FRAP tests, indicating a marginally higher antioxidant capacity. Specialized studies have demonstrated the antioxidant qualities of plants at IC50 values of 93.14 ± 0.93 μg/mL, 75.51 ± 0.71 μg/mL, and 73.26 ± 0.93 μg/mL (*H. sabdariffa*), values particularly attributed to anthocyanins/flavonoids [20,28,51,55], while Magfiroh et al. (2023) detected an IC50 value of 288.85 g/mL for chrysanthemums [58]. Red flowers are rich in phenolic compounds, according to Park et al. (2018), with values reaching up to 17.31 mg/g [46]. *P. grandiflora* shows the lowest values for all analyzed parameters, suggesting a reduced antioxidant capacity compared to the other species.

Other studies have shown that bioactive compounds in red flowers can lead to alterations in sleep [26], or have therapeutic properties [59]. Another quality of the presence of bioactive compounds in red flowers is their ability to inhibit both Gram-positive and Gram-negative bacteria as well as molds.

Analyzing the correlation between the mechanisms of action of the proposed activities and the main phenolic compounds associated with these activities, it can be stated that a high TPC (Total Phenolic Content) is associated with better antioxidant activity (ABTS and FRAP) and with higher concentrations of anthocyanins, confirming that phenolics play a key role in the antioxidant properties of plants. Anthocyanins contribute, but not exclusively, to antioxidant activity. For example, *H. sabdariffa* has a moderate concentration of TPC and anthocyanins, yet its antioxidant activity is closer to that of other species with higher TPC. The antioxidant activities (ABTS and FRAP) of the analyzed species are considerably lower than the ascorbic acid standard, highlighting the importance of other factors in assessing antioxidant potential.

Species with higher Total Phenolic Content (TPC) and anthocyanins tend to exhibit stronger antioxidant activity. *R. hybrida* L. and *M. liliiflora* are promising species for applications in antioxidant products, due to their combination of phenolic compounds and high activities. Correlating phenolic compounds with antioxidant activity, it is observed that the species *R. hybrida* L., which has the highest concentration of total phenols (107.49 mg GAE/g d.w.), also exhibits the highest antioxidant activity in ABTS (49.89 μmol TE/g d.w.) and FRAP (48.68 μmol TE/g d.w.). However, the DPPH activity is slightly lower compared to other species, suggesting that phenols are not the sole determinants of the antioxidant effect. *P. grandiflora* has the lowest total phenol content (41.61 mg GAE/g d.w.) and correspondingly, the lowest antioxidant activity across all methods (DPPH: 15.68 mg/mL, ABTS: 21.17 μmol TE/g d.w., FRAP: 22.18 μmol TE/g d.w.).

The influence of specific phenolic compounds on antioxidant activity is evident from the results obtained. Predominant phenolic acids (such as benzoic, caffeic, and ferulic) show high concentrations of benzoic acid (13,421.74 ± 56.26) and synergize with other compounds, contributing to the increased antioxidant activity in *R. hybrida* L. Species with lower phenolic acids, like *P. grandiflora*, exhibit reduced antioxidant activities. Flavonoids, rutin and quercetin, present in high concentrations in *M. liliiflora* and *R. hybrida* L., explain their better antioxidant activity. Antioxidant activity is directly proportional to the total phenol content (TPC), especially for the ABTS and FRAP methods.

Specific phenolic compounds, such as flavonoids and phenolic acids, significantly contribute to the antioxidant capacity, hence *R. hybrida* L. has a complex phenolic profile which determines maximum antioxidant activity, while *P. grandiflora*, with the lowest phenol content, shows the weakest antioxidant properties. Differences between the DPPH, ABTS, and FRAP methods indicate that antioxidant mechanisms can be different (free radical neutralization versus oxidant reduction). There is a significant correlation between the biochemical characteristics of the extracts and antibacterial activity. High levels of TPC and anthocyanins contribute to high antibacterial activity against both Gram-positive and Gram-negative bacteria. Lower levels of antioxidants compared to *R. hybrida* or *M. liliiflora* are reflected in moderate antibacterial efficiency. Reduced activity against *P. fluorescens* and *P. mirabilis* (MIC: ≥40 mg/mL) may support efficient antibacterial activity, especially against Gram-positive bacteria. Extracts from *P. grandiflora* exhibit weak activity for all tested bacteria (MIC: ≥20 mg/mL) correlated with reduced levels of antioxidants and phenolics. Good performance for *S. aureus* and *E. coli* (MIC: 10 mg/mL; MBC: 20 mg/mL) is presented by *R. hybrida*; however, it has reduced activity against *P. fluorescens* and *P. mirabilis* (MIC: ≥40 mg/mL). High levels of antioxidants support significant antibacterial activity against sensitive bacteria.

A general correlation shows that extracts with high TPC (*M. liliiflora* and *R. hybrida*) have exhibited the best results against *S. aureus* and *E. coli*, while low levels of TPC (*P. grandiflora*) correspond to weak antibacterial activity. ABTS and FRAP activities are higher for *R. hybrida* and *M. liliiflora*, reflecting a positive correlation with effectiveness against Gram-positive bacteria. Higher levels of anthocyanins and antioxidant efficiency (DPPH) are associated with better antibacterial activity (*R. hybrida*, *M. liliiflora*). Antibacterial efficiency is directly influenced by the chemical composition of the extracts. Species with high TPC, anthocyanins, and antioxidant activity are more effective against both Gram-positive and Gram-negative bacteria, but less effective against resistant strains such as *P. fluorescens*.

**Figure 3:**
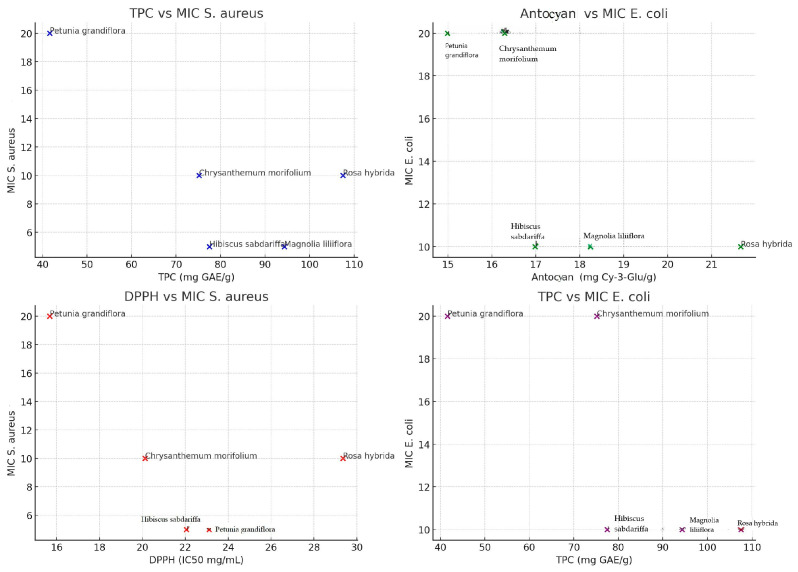
Scatter plots showing the correlations between biochemical variables and antibacterial activity.

Observing the scatter plots (Figure 3), it is noted that plants with higher Total Phenolic Content (TPC) tend to have a lower Minimum Inhibitory Concentration (MIC), suggesting increased antibacterial efficiency. Elevated levels of anthocyanins are associated with better antibacterial efficacy against *E. coli*. Antioxidant activity (lower DPPH) appears to be positively correlated with antibacterial efficiency against *S. aureus*, and a similar trend indicates that higher TPC is associated with lower MIC values for *E. coli*. Following the relationship between TPC (total polyphenols) and antibacterial activity (MIC *S. aureus* and MIC *E. coli*), it is observed that plants with higher TPC content (*R. hybrida* and *M. liliiflora*) tend to have lower MIC values, indicating better antibacterial activity against both bacterial strains. Plants with low TPC (*P. grandiflora*) exhibit weaker antibacterial efficiency (higher MIC). It can be stated that TPC is an important indicator of antibacterial activity, suggesting that polyphenols significantly contribute to the ability to inhibit bacterial growth.

Tracking the relationship between total anthocyanins and antibacterial activity reveals that plants with higher anthocyanin content (*R. hybrida*: 21.66 mg Cy-3-Glu/g) demonstrated better antibacterial activity than those with fewer anthocyanins (*P. grandiflora*: 14.99 mg Cy-3-Glu/g), and the effectiveness against *E. coli* appears more sensitive to anthocyanin levels compared to *S. aureus*. The high level of anthocyanins contributes to antibacterial activity, possibly due to mechanisms involving bacterial cell wall disruption through oxidative reactions.

Following the relationship between antioxidant activity (DPPH, ABTS, FRAP) and MIC, it can be observed that extracts with lower DPPH values (greater antioxidant efficiency) tend to have more antibacterial effect. For example, *R. hybrida* and *M. liliiflora* exhibit superior antioxidant and antibacterial activity. Higher values of antioxidant activity (μmol TE/g) coincide with better antibacterial activity, especially in *R. hybrida* (ABTS: 49.89 μmol TE/g; FRAP: 48.68 μmol TE/g), while extracts with low antioxidant activity (*P. grandiflora*) also had reduced antibacterial efficiency. The correlation between these aspects leads to the hypothesis that antioxidant activity plays a role in antibacterial activity, through the generation of reactive oxygen species that can affect the bacterial cell wall. This analysis also reveals some discrepancies and exceptions: *C. morifolium* has relatively good TPC and anthocyanin content, but moderate antibacterial efficiency, suggesting that other bioactive compounds may influence the results. *H. sabdariffa* has TPC and anthocyanins comparable to *R. hybrida*, yet its antibacterial efficiency is lower, indicating a possible difference in specific chemical composition or weaker synergies between antioxidants. From this analysis, direct correlations can be established: TPC and anthocyanins strongly correlate with antibacterial efficiency against Gram-positive (*S. aureus*) and Gram-negative bacteria (*E. coli*), and antioxidant activity (ABTS and FRAP) further contributes to antibacterial activity.

Among the species, there are several distinctions, such that it can be stated that *R. hybrida* and *M. liliiflora* are the most promising species due to their high values in TPC, anthocyanins, and antioxidant activity, while *P. grandiflora* has the weakest potential, limited by its poorer chemical composition.

The values presented in Figure 3 lead to a series of Pearson correlations involving the relationships between biochemical variables and antibacterial activity (MIC).

For TPC (total polyphenols), the following correlations result:

- TPC vs. MIC *S. aureus*:
  ○ r = −0.73, *p* = 0.16
  ○ Moderate to strong negative correlation, but not statistically significant.
- TPC vs. MIC *E. coli*:
  ○ r = −0.77, *p* = 0.13
  ○ Moderate to strong negative correlation.

For anthocyanins, the results are as follows:

- Anthocyanins vs. MIC *S. aureus*:
  ○ r = −0.42, *p* = 0.48
  ○ Weak negative correlation.
- Anthocyanins vs. MIC *E. coli*:
  ○ r = −0.72, *p* = 0.17
  ○ Moderate negative correlation.

Antioxidant activity (DPPH) shows the following correlations:

- DPPH vs. MIC *S. aureus*:
  ○ r = −0.57, *p* = 0.32
  ○ Moderate negative correlation, not statistically significant.
- DPPH vs. MIC *E. coli*:
  ○ r = −0.76, *p* = 0.13
  ○ Moderate to strong negative correlation.

In this case, it can be stated that negative correlations are present where the negative values of the coefficients suggest that a higher content of polyphenols, anthocyanins, and antioxidant activity is associated with lower MIC values (better antibacterial activity); none of the correlations are statistically significant (*p* > 0.05), which indicates a possible insufficiency of data (small number of observations).

Although the *p*-values do not indicate statistical significance, the general trends show a positive relationship between biochemical compounds and antibacterial activity.

**Figure 4:**
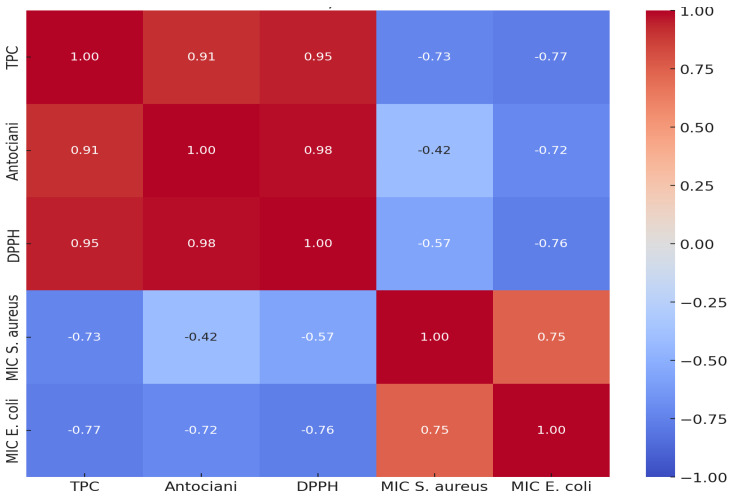
Correlation matrix between biochemical variables and antibacterial activity.

In Figure 4, significant negative correlations are observed between TPC, DPPH, and MIC values for the bacteria *S. aureus* and *E. coli*, indicating an association between antioxidant activity and antibacterial efficiency. The linear regression between TPC and MIC illustrates the linear relationship between TPC content and antibacterial activity through regression. Thus, the MIC for *S. aureus* presents a model with an R^2^ coefficient of 0.531, suggesting that approximately 53% of the variation in MIC values can be explained by TPC. The coefficient for TPC is −0.1799, indicating a decrease in MIC by 0.18 units for every increase of 1 mg GAE/g in TPC, with a *p*-value of 0.163 suggesting a statistically non-significant trend. Observing MIC for *E. coli*, the model has an R^2^ coefficient of 0.587, indicating that 58% of the variation in MIC values can be explained by TPC. The coefficient for TPC is −0.1691, indicating a decrease in MIC by 0.17 units for each increase of 1 mg GAE/g in TPC, and a *p*-value of 0.131 indicates a relationship that is not statistically significant but shows a clear trend. Consequently, it can be stated that TPC strongly influences MIC values, suggesting that polyphenols are major factors in antibacterial activity, but the relationship is not fully statistically significant. Although statistical tests do not confirm the significance of the relationships due to the small sample size (five observations), the observed trends support the hypothesis of a significant chemical influence on antibacterial activity.

Thus, the studies conducted by Arredondo-Valdes et al. (2020), Chang et al. (2021), Manandhar et al. (2019), Jacobo-Salcedo et al. (2011), Hodaei et al. (2020), and Panaitescu et al. (2017), demonstrate the antibacterial and antifungal activities of extracts from magnolia, chrysanthemums, and hibiscus [21,32,41,60,61,62,63]. These have proven effective against strains of *E. coli*, *Enterobacter cloacae*, *S. aureus*, *Pseudomonas syringae*, *X. vesicatoria*, *Enterococcus faecalis*, *A. flavus*, and *Candida albicans*.

## 5. Conclusions

Following the applied extraction procedures, it is observed that *R. hybrida* L. stands out with the highest quantity and concentration of extract, suggesting a higher potential to extract active compounds. *P. grandiflora* shows the lowest values for both parameters, indicating a lower yield. *M. liliiflora* and *H. sabdariffa* have moderate quantities, while *C. morifolium* presents lower values compared to the other species. *R. hybrida* L. is notable for the most significant indicators in almost all testing categories (phenolics, anthocyanins, DPPH, ABTS, FRAP), suggesting a high antioxidant potential, followed by *M. liliiflora*, while *P. grandiflora* shows the lowest values compared to the extracts studied. Phenolic compounds are marked by concentrations of benzoic acid, which is present in large amounts in all species, with the highest values in *R. hybrida* L. and *H. sabdariffa*. Rutin and quercetin, two important flavonoids, are well-represented in all plants, with peak values in *M. liliiflora* and *H. sabdariffa*. Each plant has a unique profile of bioactive compounds, with variations depending on the species analyzed.

Following the analysis of antibacterial activity, it can be concluded that *H. sabdariffa* and *R. hybrida* L. are the most effective extracts, exhibiting extensive characterized by low MIC and MBC values, which are indicative of strong antibacterial activity. *C. morifolium* and *P. grandiflora* show limited activity against certain bacteria, with no action against species such as *P. fluorescens*. The reference antibiotic generally has the lowest MIC and MBC values, but some plants, especially *H. sabdariffa* and *R. hybrida* L., demonstrate comparable efficacy. Research has shown that the studied extracts are a potential source of natural antioxidants and antibacterials. These properties can be extrapolated to the food, pharmaceutical, or medical industries.

## Figures and Tables

**Table 1:** Values of extract (mg) and final concentrations identified in samples from *M. liliiflora*, *C. morifolium*, *H. sabdariffa*, *P. grandiflora*, *R. hybrida* L., expressed in mg/mL (mass concentrations).

Sample		Sample 1	Sample 2	Sample 3	Average
*M. liliiflora*	Dry Extract (mg)	54.13	61.27	49.87	55.09
	Extract Concentration (mg/mL)	5.41	6.12	4.98	5.50
*C. morifolium*	Dry Extract (mg)	37.99	43.23	40.14	40.45
	Extract Concentration (mg/mL)	3.79	4.32	4.01	4.04
*H. sabdariffa*	Dry Extract (mg)	44.16	45.57	45.81	45.18
	Extract Concentration (mg/mL)	4.41	4.55	4.58	4.51
*P. grandiflora*	Dry Extract (mg)	26.29	32.22	29.11	29.20
	Extract Concentration (mg/mL)	2.62	3.22	2.91	2.92
*R. hybrida* L.	Dry Extract (mg)	68.25	70.19	74.85	71.09
	Extract Concentration (mg/mL)	6.82	7.01	7.48	7.10

**Table 2:** Variation in polyphenols, total anthocyanins, and antioxidant activity of extracts from *M. liliiflora*, *C. morifolium*, *H. sabdariffa*, *P. grandiflora*, and *R. hybrida* L.

	TPC mg GAE/g d.w.	Total Anthocyanins mg Cy-3-Glu/g d.w.	DPPH IC50 mg/mL	ABTS μmol TE/g d.w.	FRAP μmol TE/g d.w.
*M. liliiflora*	94.34 ± 0.21	18.24 ± 0.11	23.11 ± 0.17	42.19 ± 0.11	45.71 ± 0.22
*C. morifolium*	75.17 ± 0.16	16.29 ± 0.11	20.12 ± 0.11	38.11 ± 0.12	41.23 ± 0.11
*H. sabdariffa*	77.44 ± 0.16	16.98 ± 0.13	22.05 ± 0.12	40.19 ± 0.19	39.32 ± 0.17
*P. grandiflora*	41.61 ± 0.11	14.99 ± 0.13	15.68 ± 0.10	21.17 ± 0.12	22.18 ± 0.15
*R. hybrida* L.	107.49 ± 0.19	21.66 ± 0.18	29.35 ± 0.17	49.89 ± 0.24	48.68 ± 0.23
Ascorbic acid	-			21.33 ± 1.13 mmol TE/g d.w.	22.27 ± 1.21 mmol TE/g d.w.

TPC: total polyphenol content; GAE: gallic acid equivalents; Cy-3-Glu: cyanidin-3-glucoside equivalents; DPPH free radical scavenging IC 50; ABTS: radical cation scavenging assay; FRAP: ferric-reducing antioxidant power; TE: Trolox equivalents. Values are expressed as means ± SD.

**Table 3:** Phenolic and Flavonoid Compounds Quantified in Extracts from *M. liliiflora*, *C. morifolium*, *H. sabdariffa*, *P. grandiflora*, *R. hybrida* L., expressed in μg/mL (average values of three determinations with standard error).

Compound	*M. liliiflora*	*C. morifolium*	*H. sabdariffa*	*P. grandiflora*	*R. hybrida* L.
Benzoic Acid	10,873.22 ± 25.04	11,209.12 ± 55.19	12,009.37 ± 50.57	10,568.93 ± 43.22	13,421.74 ± 56.26
Caffeic Acid	21.13 ± 1.31	87.26 ± 2.05	234.59 ± 15.27	99.98 ± 10.13	207.43 ± 11.54
Chlorogenic Acid	5683.35 ± 26.01	3794.48 ± 29.67	5544.34 ± 27.55	2272.32 ± 11.18	3896.51 ± 12.01
Ellagic Acid	nd	20.22 ± 1.21	234.77 ± 15.71	nd	54.45 ± 1.11
Ferulic Acid	1065.77 ± 19.58	643.88 ± 19.22	983.26 ± 17.04	197.48 ± 5.21	1012.66 ± 18.92
Gallic Acid	298.29 ± 8.81	562.55 ± 15.51	884.44 ± 17.11	109.99 ± 5.33	709.99 ± 16.13
m-Coumaric Acid	5.66 ± 0.59	29.09 ± 2.72	31.31 ± 2.12	nd	12.12 ± 0.94
p-Coumaric Acid	562.55 ± 10.16	389.27 ± 11.23	788.33 ± 11.63	656.72 ± 15.01	1022.95 ± 18.55
p-Hydroxybenzoic Acid	nd	41.13 ± 1.54	nd	18.96 ± 0.59	66.18 ± 1.01
Rosmarinic Acid	nd	2.33 ± 0.30	109.99 ± 3.34	nd	99.88 ± 3.45
Salicylic Acid	nd	1.22 ± 0.03	19.72 ± 0.59	nd	27.91 ± 1.17
Sinapic Acid	nd	nd	0.27 ± 0.00	nd	nd
Syringic Acid	893.27 ± 15.16	562.29 ± 14.14	1001.10 ± 17.34	873.88 ± 15.11	1231.55 ± 17.22
Trans-Cinnamic Acid	0.26 ± 0.00	nd	45.62 ± 0.01	12.97 ± 0.01	34.52 ± 0.01
Vanillic Acid	129.92 ± 2.52	197.88 ± 3.25	243.45 ± 5.95	308.75 ± 5.55	527.87 ± 6.23
Rutin	3456.32 ± 19.15	2996.48 ± 22.07	1999.57 ± 17.31	2341.73 ± 19.44	1912.31 ± 15.16
Quercetin	892.88 ± 5.01	784.59 ± 6.28	1001.72 ± 6.26	777.15 ± 5.71	999.15 ± 2.37
(+)-Catechin	108.95 ± 4.54	77.94 ± 2.23	93.26 ± 4.58	102.21 ± 5.18	128.98 ± 5.33
Luteolin	67.97 ± 2.31	112.07 ± 7.61	65.46 ± 2.19	99.17 ± 3.27	134.83 ± 5.22
Apigenin	105.03 ± 4.35	198.45 ± 9.11	19.19 ± 0.06	97.79 ± 4.22	77.03 ± 3.19
Total	23,291.35	21,710.25	25,309.49	18,435.82	25,578.06

nd—not determined; values are expressed as means ± SD.

**Table 4:** Antibacterial activity, Minimum Inhibitory Concentration (MIC) (mg/mL) and Minimum Bactericidal Concentrations (MBC) (mg/mL) of extracts from *M. liliiflora*, *C. morifolium*, *H. sabdariffa*, *P. grandiflora*, *R. hybrida* L.

Bacterial Strains	Activity	*M. liliiflora*	*C. morifolium*	*H. sabdariffa*	*P. grandiflora*	*Rosa* *hybrida* L.	Ampicillin
*S. aureus* ATCC 33862	AA	++++	+++	++++	++	+++	+++++
	MIC	5	10	5	20	10	<1.5
	MBC	10	20	10	40	20	1.5
*E. coli* ATCC 25922	AA	+++	++	+++	++	+++	+++++
	MIC	10	20	10	20	10	<1.5
	MBC	20	40	20	40	20	1.5
*P. fluorescens* ATCC 13525	AA	+	+	+	−	−	++++
	MIC	40	40	40	−	−	<1.5
	MBC	>40	>40	>40	−	−	1.5
*P. mirabilis* ATCC 12453	AA	++	+	++	+	+	++++
	MIC	20	40	20	40	40	<1.5
	MBC	40	>40	40	>40	>40	1.5

AA—Antibacterial Activity; MIC—Minimum Inhibitory Concentration (mg/mL); MBC—Minimum Bactericidal Concentrations (mg/mL). +++++ very strong activity (over 25 mm)/++++ strong activity (21–24 mm), +++ moderate activity (15–20 mm), ++ reduced activity (10–14 mm), + very reduced activity (7–9 mm), − no activity.

## Data Availability

Data are contained within the article.
